# Potential prognostic impact of left-ventricular global longitudinal strain in analysis of whole-heart myocardial mechanics in nonischemic dilated cardiomyopathy

**DOI:** 10.1007/s10554-024-03184-x

**Published:** 2024-07-03

**Authors:** Karolina Mėlinytė-Ankudavičė, Karolina Marcinkevičienė, Grytė Galnaitienė, Paulius Bučius, Tomas Lapinskas, Eglė Ereminienė, Gintarė Šakalytė, Renaldas Jurkevičius

**Affiliations:** 1 Department of Cardiology, Medical Academy, Lithuanian University of Health Sciences, 44307 Kaunas, Lithuania; 2 Institute of Cardiology, Lithuanian University of Health Sciences, 50162 Kaunas, Lithuania; 3 Department of Radiology, Medical Academy, Lithuanian University of Health Sciences, 44307 Kaunas, Lithuania

**Keywords:** Heart failure, Non-ischemic dilated cardiomyopathy, Early primary outcomes; whole-heart mechanics, Cardiac magnetic resonance, Global longitudinal strain

## Abstract

Cardiac magnetic resonance (CMR) feature-tracking (FT) has an important diagnostic role in non-ischemic dilated cardiomyopathy (NIDCM). To date, the relationship between whole-heart myocardial mechanics by CMR and early primary outcomes in NIDCM has not been elucidated. patients with NIDCM were eligible for this study. CMR-FT was used to analyze whole-heart myocardial mechanics. The primary outcomes were a composite of heart failure (HF) death, heart transplantation (HT), and hospitalization for HF worsening (WHF) after 1-year since diagnosis. 98 patients were included. During a 1-year follow-up, a worse prognosis occurred in 32 patients (30 hospitalizations for WHF, 8 deaths, and 3 HT). The left ventricular (LV) global longitudinal systolic strain (GLS), left ventricular global circumferential strain (LV GCS), strains of right ventricle and both atria were significantly reduced in patients with events vs. those without (GLS − 8.0 ± 3.4 vs. − 12.1 ± 4.5, *p* < 0.001; GCS − 13.0 ± 6.4 vs. − 18.3 ± 7.1, *p* < 0.001; right ventricular (RV) GLS − 12.1 ± 4.9 vs. − 17.4 ± 6.4, *p* < 0.001; left atrial longitudinal strain 7.5 ± 3.8 vs. 15.1 ± 12.3, *p* < 0.001; right atrial longitudinal strain 11.0 ± 6.7 vs. 17.2 ± 8.0, *p* < 0.001). Left ventricular ejection fraction (LVEF) was significantly higher in patients with better prognosis (22.7 ± 8.7 vs. 33.56 ± 10.4, *p* < 0.001). Multivariate regression analysis revealed LV GLS as an independent predictor of a worse prognosis (OR 0.787, CI 95% 0.697–0.890, *p* < 0.001). reduction of LV GLS showed the strongest predictive value for the composite outcome of WHF, HT, and HF death.

**Graphical abstract:**

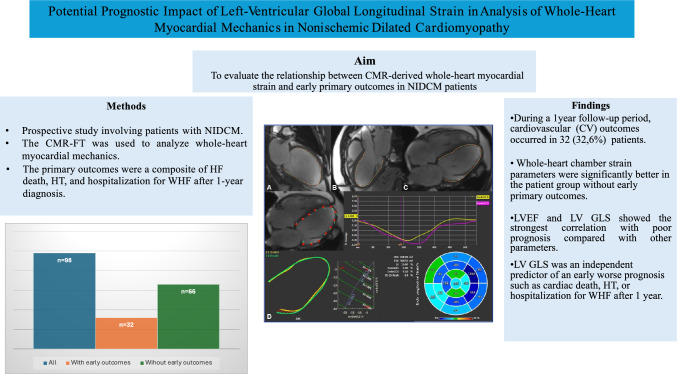

## Introduction

NIDCM is a severe form of primary myocardial disease characterized by dilatation and dysfunction of the left or both ventricles. Despite the recent progress in medical care, progressive HF with a poor prognosis is related with NIDCM [1]. Current diagnostic methods rely on a combination of clinical assessment, family history, and genetics alongside imaging methods [2]. NIDCM often features an extended sub-clinical phase–a period where symptoms and myocardial dysfunction may not be present [3], this can potentially lead to a delay in accurate diagnosis and timely prescription of treatment. Thus, more sensitive tools are needed for detailed risk evaluation.

CMR has become the most useful and accurate non-invasive technique for assessing cardiac structure and function [4]. CMR has recently been proven as a reliable method for determining functional myocardial deformation parameters using FT, and may offer greater clinical value compared to LVEF measurements alone [5]. Previous small and large multicenter studies with long term follow up (up to 4–6 years) demonstrated the independent and incremental predictive value of CMR derived LV GLS for both ischemic and nonischemic dilated cardiomyopathy patients [6, 7]. Despite the surge in reports about this issue, most of the studies that investigated patients with NIDCM only limited analysis to standalone LVEF, LV, or RV deformation parameters [7, 9, 11]. Regarding risk stratification using the FT-derived mechanics for patients with NIDCM, there are no studies that analyzed whole-heart strain parameters. In this context, we aimed to perform a study assessing the relationship between CMR-derived whole-heart myocardial strain and early primary outcomes in NIDCM patients.

## Methods

### Study population

This was a prospective study involving patients with NIDCM. The diagnosis of NIDCM was made according to the latest European Society of Cardiology (ESC) document [2]. The exclusion criteria for the study were:

- an ischemic coronary disease (more than 50% coronary artery occlusion by invasive or cardiac computed tomography coronary angiography);
- primary valvular heart disease;
- chronic severe kidney disease;
- poor CMR quality;
- inflammatory myocardial disease;
- tachycardia-induced HF;
- previous pulmonary embolism;
- peripartum cardiomyopathy;
- toxic damage;
- over 18 years of age.

of 110 patients with a diagnosis of NIDCM, we excluded 12 because their CMR image quality was inappropriate for analysis, resulting in a final sample size of 98 patients. Basic clinical characteristics, laboratory results, and electrocardiography were recorded in standard form. All participants gave written informed consent before enrollment. The study was approved by the local institutional ethics committee.

### Follow-up and endpoints

The study consisted of two phases: during the first phase, patients were enrolled and examined for the first time and diagnosed with NIDCM (patients without chronic or worsening HF); during the second phase, early primary outcomes were evaluated after 1-year follow-up. During follow-up, all patients were treated with an optimal HF treatment according to chronic HF guidelines [12]. Information about the presented adverse events was collected from medical records or patients were invited for a follow-up visit. The early primary outcomes were cardiac death, heart transplantation, and hospitalization for WHF at one year. WHF was defined according to current recommendations by the American College of Cardiology (patients admitted to hospital with decompensated HF requiring treatment with intravenous HF drugs) [13].

### CMR protocol and strain analysis

The CMR analysis was conducted with a 3.0-T magnetic resonance imaging scanner (MAGNETOM Skyra, Siemens Healthcare, Erlangen, Germany) using an 18-channel cardiac coil and electrocardiogram gating. Cine images were acquired using standard balanced steady-state free precession (bSSFP) sequences in long axes (2-, 3-, and 4-chamber) and the stack of short axis (covering entire ventricles) views during an expiratory breath hold. The CMR images were transferred to an off-line workstation with CMR post-processing software Medis Suite 3.1 (Medis Medical Imaging, Leiden, The Netherlands), and the data were analyzed by a trained, blinded observer.

LV and RV volumes, LVEF, right ventricular ejection fraction (RVEF), and LV mass were evaluated from short-axis cine images using standard volumetric techniques [14]. CMR strain analysis was performed using the FT application QStrain (Medis Suite 3.1, Medis Imaging, Leiden, The Netherlands). The endocardial borders of all cardiac chambers were traced manually in end-systole and an automated tracking algorithm was used, further manual adjustments were performed when needed [15].

**Fig. 1:**
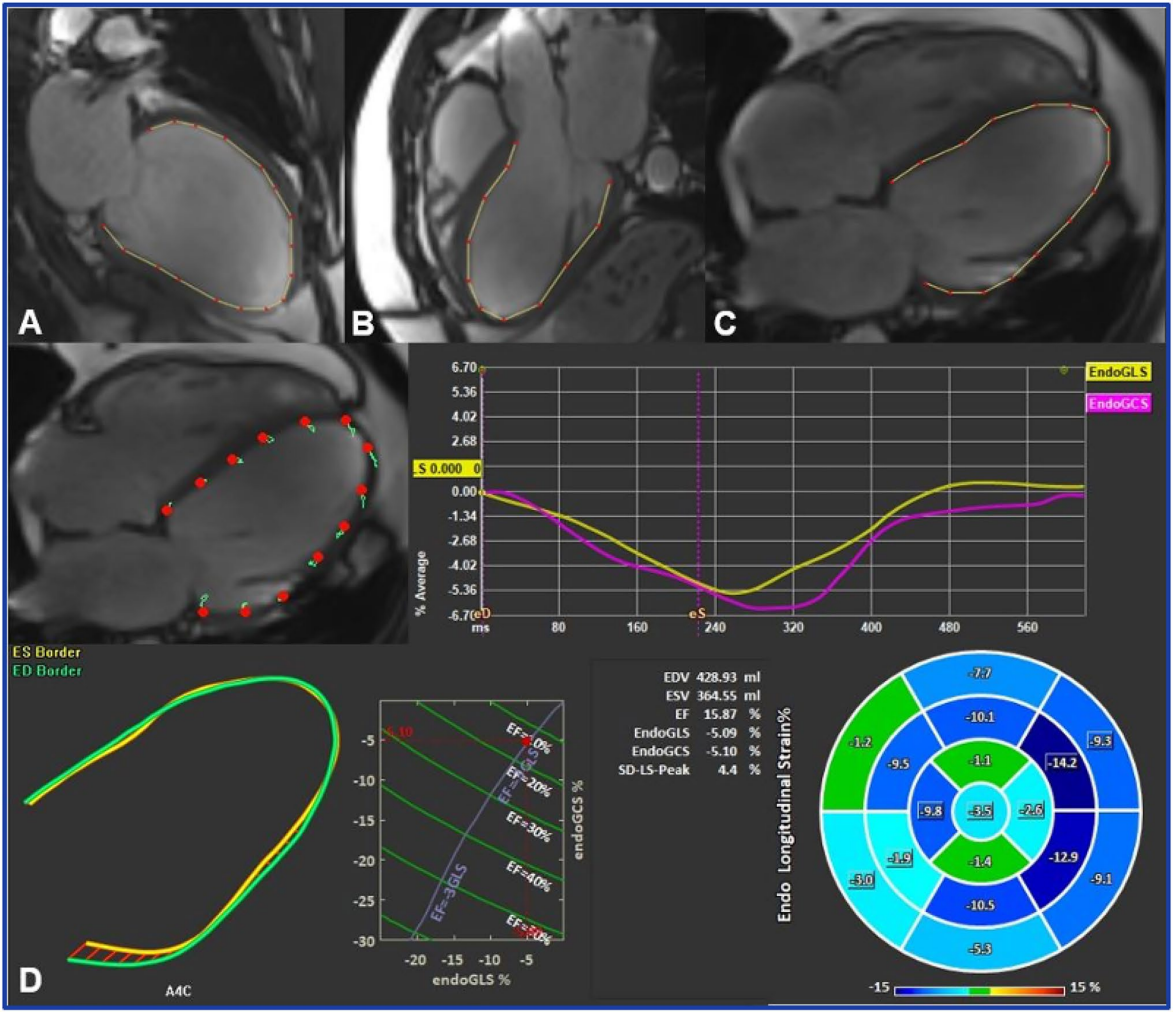
Example of left ventricular global longitudinal strain assessment using feature tracking software. Endocardial borders were delineated on long-axis two-(**A**), three-(**B**), and four-chamber (**C**) SSFP cine images in end-systole and end-diastole. The final automatic calculation was performed by the software: the schematic picture shows minimal myocardium movement and the average GLS of all 17 cardiac segments in this case was − 5.09 (**D**)

LV GLS and GCS were derived by averaging the peak strain values of individual segments using a 17 segment model. The LV GLS was calculated from 2-, 3-, and 4-chamber long-axis views (Fig. 1).

Mean T1 map values were measured on T1 mapping short-axis images by drawing a region of interest (ROI) in the septum at LV basal, mid, and apical planes. This was performed both before and after gadolinium-based contrast agent administration.

For myocardial extracellular volume (ECV) evaluation, a ROI in the center of the blood pool in the native and the post-contrast T1 mapping images was drawn, excluding papillary muscles and trabeculae. Hematocrit level was determined for each patient from a venous blood sample less than 24 h before the CMR examination.

**Fig. 2:**
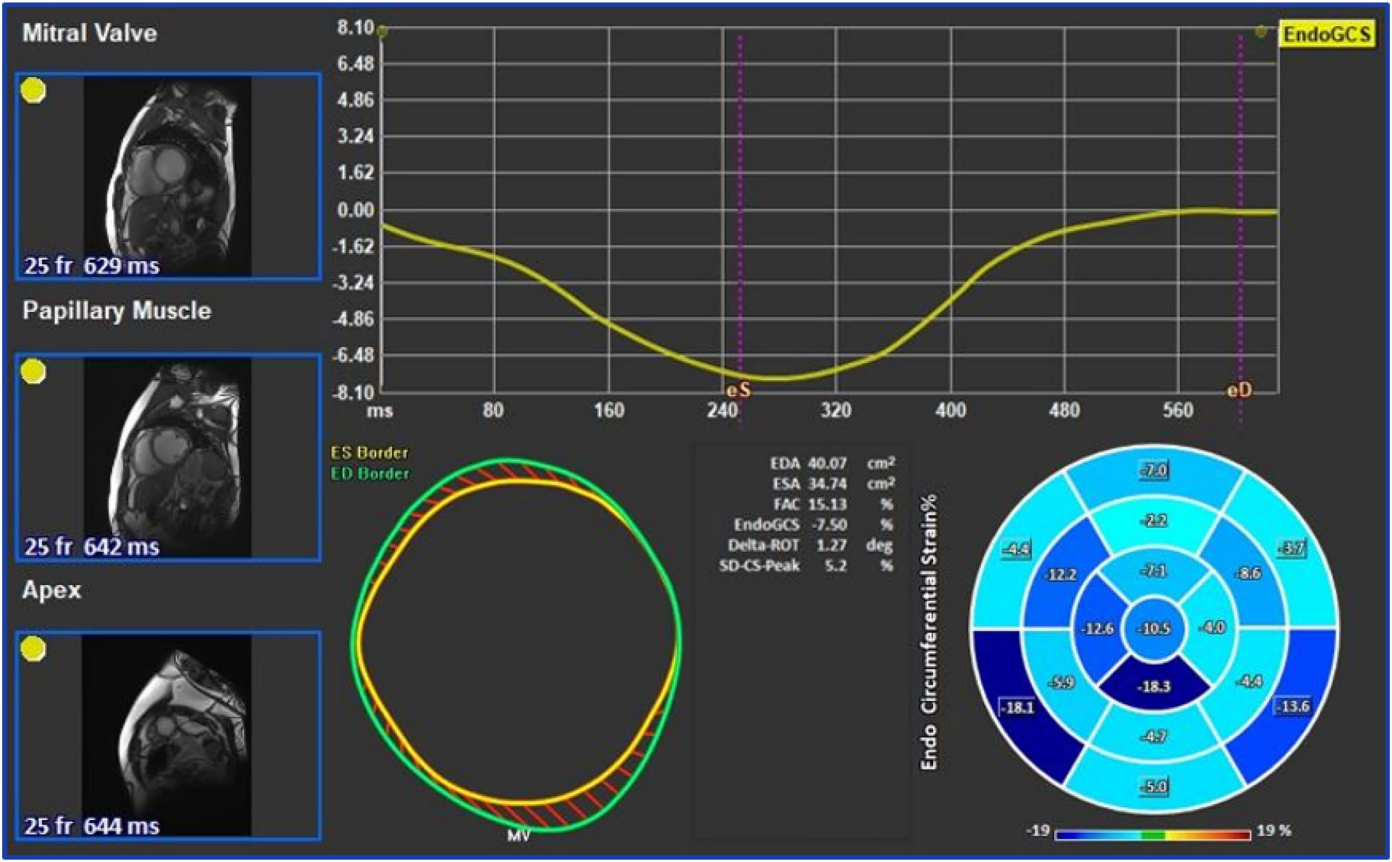
Left ventricular global circumferential strain assessment using feature tracking software

The LV GCS was automatically calculated from previously traced points in QMass on short-axis views at the base, mid, and apex planes during end-systole and end-diastole (Fig. 2).

**Fig. 3:**
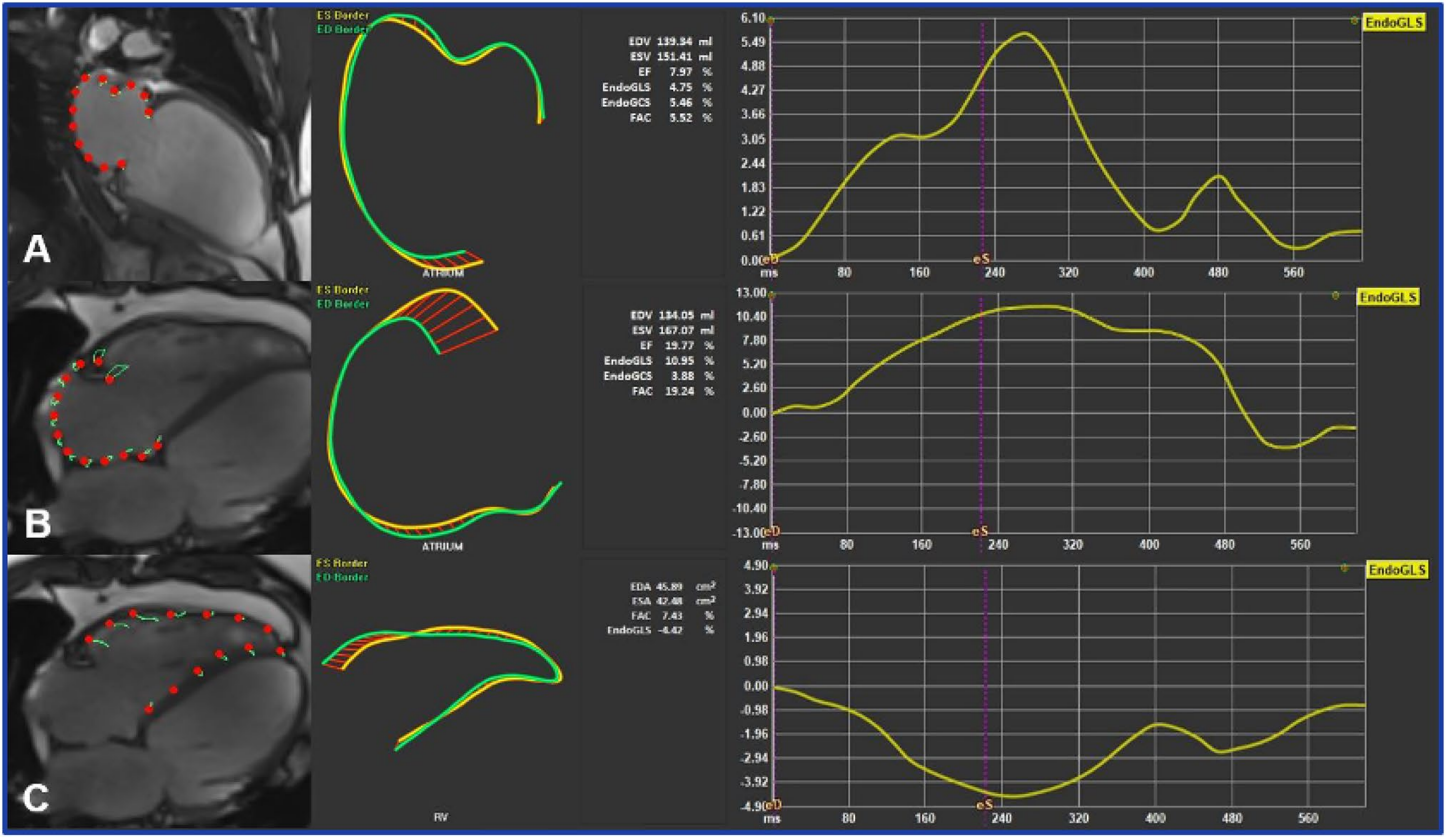
Cardiac magnetic resonance imaging-derived whole-heart myocardial mechanical parameters assessment using feature tracking software. Global longitudinal strain of the left atrium **A** right atrium **B** and right ventricle **C** was assessed by tracing endocardial borders on long-axis SSPF cine images in end-systole and end-diastole

RV and right atrial (RA) GLS were calculated from 4-chamber, while left atrial (LA) GLS was calculated from 2-chamber long-axis views (Fig. 3).

### Statistical analysis

The results were presented as means ± standard deviations (SD) or as absolute numbers and percentages. The study population was divided into two groups according to the presence of outcomes (with or without the early primary outcomes). The student’s t‐test was used to compare normally distributed parameters, and the Mann–Whitney U‐test for abnormally distributed parameters. The point-biserial coefficient of correlation R^2^ was used to assess the relationship between myocardial mechanics and morphometrics, and the presence of worse prognosis. Binary logistic regression analysis was used to determine the potential predictors of a worse prognosis. Intra- and interobserver variability was tested by using the intraclass correlation coefficient. A *p*-value of < 0.05 was considered statistically significant. Analyses were performed using SPSS version 22 (IBM, Chicago, IL, USA).

## Results

The baseline characteristics of the entire study cohort are described in Table 1. There were no significant differences in mean age, sex, risk factors (arterial hypertension, dyslipidemia, smoking etc.), and clinical characteristics (New York Heart Association (NYHA) class, atrial fibrillation etc.) in both groups (*p* > 0.05). The brain natriuretic peptide (BNP) and C-reactive protein concentrations were higher in patients with worse prognosis (1858.1 ± 963.2 vs. 791.9 ± 398.2 ng/l, *p* = 0.01; 10.9 ± 5.8 vs. 8.3 ± 5.0 g/l, *p* = 0.03). The presence of early primary outcomes in NIDCM patients was not linked to genetically identified pathogenic variants. During a 1-year follow-up period, cardiovascular (CV) outcomes occurred in 32 (32,6%) patients: 30 (93.8%) have been hospitalized for HF worsening, including 8 (25%) deaths and 3 (9.4%) heart transplants.

**Table 1:** Baseline characteristics for NIDCM patients with and without early primary outcomes

Variables	Patients with the early primary outcomes n = 32	Patients without the early primary outcomes n = 66	p-value
Age, y	49.5 ± 11.3	49.5 ± 9.5	0.98
Males, n (%)	22 (32.4)	46 (67.6)	0.55
BMI, m^2^	27.8 ± 5.5	29.2 ± 5.3	0.23
Dyslipidemia, n (%)	12 (31.6)	26 (68.4)	1.00
Arterial hypertension, n (%)	17 (30.4)	39 (69.6)	0.66
Smoking, n (%)	18 (43.9)	23 (56.1)	0.05
Genetic analysis: positive, n (%) refuse the genetic test, n (%)	5 (20) 11 (78.6)	20 (80) 3 (21.4)	< 0.001
VT, n (%)	17 (54.8)	14 (45.2)	> 0.05
Atrial fibrillation, n (%)	18 (48.6)	19 (51.4)	> 0.05
NYHA class III IV, n (%)	21 (36.2) 7 (21.9)	37 (63.8) 7 (50.0)	> 0.05
TnI, ng/l	0.6 ± 1.7	0.2 ± 0.9	0.27
CRP, g/l	10.9 ± 5.8	8.3 ± 5.0	0.03
Hs-CRP	3.0 ± 1.5	3.2 ± 3.0	0.88
NLR	3.1 ± 2.5	3.1 ± 2.5	0.97
BNP, ng/l	1858.1 ± 963.2	791.9 ± 398.2	0.01
ST2, pg/ml	36.6 ± 11.4	43.1 ± 9.6	0.67
Heart failure death, n (%)	8 (25.0)	–	–
Heart transplant, n (%)	3 (9.4)	–	–
Hospitalization for HF worsening at 1 year, n (%)	30 (93.8)	–	–

BSA—body surface area; ACE-I—angiotensin-converting enzyme inhibitor; ARB—angiotensin receptor blocker; CCB—calcium channel blocker; VT—ventricular tachycardia; LBBB—left bundle branch block; HF—heart failure; CA—coronary artery; NYHA—New York Heart Association; 6MWT—6-min walk test; Hs-CRP—high sensitivity C-reactive protein; BNP—brain natriuretic peptide; NLR—Neutrophil to Lymphocyte Ratio; TnI—troponin I. The data from the MRI parameters in NIDCM patients are summarized in Table 2. The patients with worse CV outcomes had more dilated LV (*p* = 0.016), LA area (*p* = 0.005), and RV (*p* = 0.005). There was no significant difference in measurements of RA area between the groups (*p* > 0.05). Whole-heart chambers strain parameters were significantly better in the patient group without early primary outcomes (*p* < 0.001). Patients with better prognosis had significantly higher LVEF (22.7 ± 8.7 vs. 33.56 ± 10.4, *p* < 0.001, respectively). T1 mapping and late gadolinium enhancement did not show significant connections with NIDCM patient outcomes (*p* < 0.05).

**Table 2:** MRI parameters in NIDCM patients with and without early primary outcomes

Variables	Patients with early primary outcomes n = 32	Patients without early primary outcomes n = 66	p-value
LVEDD, mm	73.6 ± 7.7	68.7 ± 11.8	0.016
LVEDDi, mm/m^2^	36.0 ± 7.9	33.4 ± 6.6	0.122
IVS, mm	9.5 ± 2.1	10.5 ± 1.9	0.032
PW, mm	8.0 ± 1.9	8.5 ± 1.9	0.231
LVEDV, ml	340.7 ± 90.6	304.9 ± 91.4	0.073
LVEDVi, mm/m^2^	165.3 ± 53.7	144.8 ± 46.4	0.147
LVESV, ml	265.9 ± 82.7	208.0 ± 86.4	0.002
LVESVi, ml/m^2^	129.4 ± 48.5	98.7 ± 42.8	0.019
LVEF,%	22.7 ± 8.7	33.56 ± 10.4	< 0.001
RVEDV, ml	212.4 ± 58.8	196.7 ± 56.8	0.586
RVESV, ml	140.3 ± 59.6	103.8 ± 53.3	0.005
LAA, m^2^	39.5 ± 14.4	31.2 ± 8.7	0.005
RAA, m^2^	31.6 ± 10.7	28.2 ± 6.9	0.104
LVGLS, %	− 8.0 ± 3.4	− 12.1 ± 4.5	< 0.001
LVGCS, %	− 13.0 ± 6.4	− 18.3 ± 7.1	< 0.001
RVLS	− 12.1 ± 4.9	− 17.4 ± 6.4	< 0.001
LALS, %	7.5 ± 3.8	15.1 ± 12.3	< 0.001
RALS, %	11.0 ± 6.7	17.2 ± 8.0	< 0.001
ECV, %	29.9 ± 3.9	34.3 ± 42.7	0.494
T1 mapping, ms	1418.8 ± 106.6	1350.6 ± 110.7	0.128
LGE, n (%)	23 (38.3)	37 (61.7)	0.786

LVEDD—left ventricular end-diastolic diameter; LVEDV—left ventricular end-diastolic volume; LVESV—left ventricular end-systolic volume; IVS—interventricular septum; PW—posterior wall; LVGLS—left ventricular global longitudinal strain; LVGCS—left ventricular global circumferential strain; LVEF—left ventricular ejection fraction; RVEDV—right ventricular end-diastolic volume; RVESV—right ventricular end-systolic volume; LAA—left atrial area; RAA—right atrial area; RVLS—right ventricular longitudinal strain; LALS—left atrial longitudinal strain; RALS—right atrial longitudinal strain; ECV—extracellular volume.

Whole-heart myocardial mechanics showed weak-moderate but significant correlations with early primary outcomes (*p* < 0.05). The parameters of left ventricular end-diastolic diameter, left ventricular end-systolic volume, right ventricular end-systolic volume, and LA area were also related with patients’ worse prognosis (*p* < 0.05). LVEF and LV GLS showed the strongest correlation with poor prognosis compared with other parameters (rs 0.457, *p* < 0.001, and rs − 0.420, respectively). However, multivariate regression analysis (Table 3) revealed that the LV GLS was an independent predictor of an early worse prognosis such as cardiac death, HT or hospitalization for WHF after one year (OR 0.787, CI 95% 0.697–0.890, *p* < 0.001).

**Table 3:** Binary logistic regression analysis for the MRI parameters related to the presence of adverse events

Parameter	OR	95% CI	*p*
LV GLS, %	0.787	0.697–0.890	< 0.001
LVEF,%	1.113	0.999–1.241	0.052
LVESVi, ml/m2	1.014	0.993–1.035	0.188
LAAi, cm2/m2	0.880	0.761–1.017	0.082
RV GLS, %	1.024	0.893–1.176	0.731
LALS, %	1.012	0.896–1.143	0.844
RALS, %	1.029	0.939–1.127	0.543

LV—left ventricular; LVESVi—left ventricular end-systolic volume index; LAAi—left atrial area index; RV—right ventricular; GLS—global longitudinal strain; LALS—left atrial longitudinal strain; RALS—right atrial longitudinal strain; CI—confidence interval; OR—odds ratio.

## Discussion

In this study, we evaluated the early primary outcomes of NIDCM and whole-heart myocardial mechanics in patients first diagnosed with NIDCM. We found that LVEF and whole-heart myocardial strain parameters were significantly better in the patient group without negative outcomes. However, the LV GLS was the only indep endent predictor of early primary outcomes after one year. Findings from this study confirmed that LV GLS offers additional prognostic insights and may have significant implications for management, clinical treatment decisions and identifying optimal parameters for assessing the clinical status during follow-up.

Traditionally NIDCM research primarily focused on assessing the mechanics and prognostic significance of individual components of the heart. However, this paper recognizes the need for a more thorough approach and represents a significant change from the usual methodology. In an effort to increase our understanding of NIDCM, this study uniquely attempts to evaluate the combined influence of all of the heart chambers on the early prognosis of this disease.

In the area of cardiac imaging, CMR has emerged as a reliable and non-invasive method for assessing myocardial tissue, providing valuable insights into cardiac structure and function. Our study did not find significant results related to T1 mapping or ECV. However, recent interest has shifted towards T1 mapping and quantifying ECV due to their potential to offer valuable insights into the presence of subtle interstitial myocardial fibrosis, diffuse changes to myocardial structure, and altered function [16–18]. Recently a growing focus appeared on the independent and incremental prognostic value of CMR FT-derived myocardial mechanics across diverse cardiac conditions [4, 5, 19]. The most attention is focused on LV GLS, showing it as a significant independent predictor for adverse outcomes in NIDCM patients. Recent studies have demonstrated the association of GLS with adverse outcomes [20, 21]. Romano et al. [6] found that the GLS was associated with mortality in both ischemic and non-ischemic dilated cardiomyopathy, independently of the LVEF and late gadolinium enhancement. The recent study by Buss et al. [7] also showed that FT-derived LV GLS was an independent predictor for a combined outcome for cardiac death and heart transplantation. These results emphasize the essential role of LV GLS and its utility in predicting a spectrum of adverse cardiovascular outcomes in patients with non-ischemic dilated cardiomyopathy. Our study results confirmed the importance of GLS.

However, it is important to mention that we evaluated whole-heart myocardial mechanics. To expand our viewpoint the examination of other heart chamber functions becomes crucial. The fact that NIDCM affects the myocardium of both ventricles and might contribute to the onset of RV dysfunction has been noted in previous studies. These studies have shown RV dysfunction as an important prognostic predictor in HF [22] as well as in NIDCM patients [23]. In our study, RV strain parameters were significantly better in the patient group without early primary outcomes, which indicates a potential prognostic value of comprehensive strain analysis. However, our study results did not show independent connections between RV measurements and outcomes.

The LA also plays a central role in cardiac performance. Its functions are intricately linked to LV function and encompass reservoir, conduit, and booster roles during different phases of the cardiac cycle. The assessment of LA longitudinal strain through various techniques, particularly CMR, has recently gained significance and has been shown to be an independent predictor of prognosis in patients with HF [24, 25]. Results of one study have revealed that lower LA reservoir and conduit strain values provided valuable prognostic insights as independent predictors of adverse clinical outcomes [26]. The often overlooked RA function has been recently explored in a study by Yangjie Li et al. The study investigated the significance of impaired RA function in patients with NIDCM [27] and RA reservoir strain, and conduit strain emerged as independent predictors of all-cause mortality even after adjusting for covariates. In our study, the assessment of LA and RA deformation parameters provided valuable insights into cardiovascular outcomes, indicating a complex link between atrial function and early prognosis in patients with severely reduced LV systolic function.

In conclusion, a comprehensive understanding of cardiac function encompasses assessments of various heart chamber strain parameters. Our study contributes to the evolving landscape of NIDCM research by emphasizing the clinical value of assessing the entire heart’s mechanics rather than individual components. We confirmed the CMR-derived LV GLS as a valuable prognostic tool, offering additional insights for clinical management and treatment decisions in patients with NIDCM.

## Conclusions

Within this study, LV GLS was found to be an independent and incremental predictor of worse outcome, which exceeded LVEF in patients with optimally treated dilated cardiomyopathy. This indicates the need to routinely include GLS in the CMR follow‐up of dilated cardiomyopathy.

## References

[1] Elliott P, Andersson B, Arbustini E, Bilinska Z, Cecchi F, Charron P, Dubourg O, Kühl U, Maisch B, McKenna WJ, Monserrat L (2008) Classification of the cardiomyopathies: a position statement from the european society of cardiology working group on myocardial and pericardial diseases. Eur Heart J 29(2):270–276. 10.1093/eurheartj/ehm342 17916581 10.1093/eurheartj/ehm342

[2] Pinto YM, Elliott PM, Arbustini E, Adler Y, Anastasakis A, Böhm M, Duboc D, Gimeno J, de Groote P, Imazio M et al (2016) Proposal for a revised definition of dilated cardiomyopathy, 367 hypokinetic non-dilated cardiomyopathy, and its implications for the clinical practice: a position 368 statement of the ESC working group on myocardial and pericardial diseases. Eur Heart J 37:1850–1858. 10.1093/eurheartj/ehv727 26792875 10.1093/eurheartj/ehv727

[3] Ugander M, Oki AJ, Hsu LY, Kellman P, Greiser A, Aletras AH et al (2012) Extracellular volume imaging by magnetic resonance imaging provides insights into overt and sub-clinical myocardial pathology. Eur Heart J 33:1268–1278. 10.1093/eurheartj/ehr481 22279111 10.1093/eurheartj/ehr481 PMC3350985

[4] De Smet K, Verdries D, Tanaka K, De Mey J, De Maeseneer M (2012) MRI in the assessment of non ischemic myocardial diseases. Eur J Radiol 81:1546–1548. 10.1016/j.ejrad.2011.02.012 21392911 10.1016/j.ejrad.2011.02.012

[5] Pedrizzetti G, Claus P, Kilner PJ, Nagel E (2016) Principles of cardiovascular magnetic resonance feature tracking and echocardiographic speckle tracking for informed clinical use. J Cardiovasc Magn Reson 18:51. 10.1186/s12968-016-0269-7 27561421 10.1186/s12968-016-0269-7 PMC5000424

[6] Romano S, Judd RM, Kim RJ et al (2018) Feature-tracking global longitudinal strain predicts death in a multicenter population of patients with ischemic and nonischemic dilated cardiomyopathy incremental to ejection fraction and late gadolinium enhancement. JACC Cardiovasc Imagin 11(10):1419–1429. 10.1016/j.jcmg.2017.10.024 10.1016/j.jcmg.2017.10.024 PMC6043421 29361479

[7] Buss SJ, Breuninger K, Lehrke S, Voss A, Galuschky C, Lossnitzer D, Andre F, Ehlermann P, Franke J, Taeger T et al (2015) Assessment of myocardial deformation with cardiac magnetic resonance strain imaging improves risk stratification in patients with dilated cardiomyopathy. Eur Heart J Cardiovasc Imagin 16:307–315. 10.1093/ehjci/jeu181 10.1093/ehjci/jeu181 25246506

[8] Park JJ, Park JB, Park JH, Cho GY (2018) Global longitudinal strain to predict mortality in patientswith acute heart failure. J Am Coll Cardiol 71:1947–1957. 10.1016/j.jacc.2018.02.064 29724346 10.1016/j.jacc.2018.02.064

[9] Morris DA, Otani K, Bekfani T, Takigiku K, Izumi C, Yuda S, Sakata K, Ohte N, Tanabe K, Friedrich K et al (2014) Multidirectional global left ventricular systolic function in normal subjects and patients with hypertension: multicenter evaluation. J Am Soc Echocardiogr 27:493–500. 10.3390/jcdd10100410 24582162 10.1016/j.echo.2014.01.017

[10] Raafs AG, Boscutti A, Henkens MT, Van Den Broek WW, Verdonschot JA, Weerts J, Stolfo D, Nuzzi V, Manca P, Hazebroek MR, Knackstedt C (2022) Global longitudinal strain is incremental to left ventricular ejection fraction for the prediction of outcome in optimally treated dilated cardiomyopathy patients. J Am Heart Assoc 11:e024505. 10.1161/JAHA.121.024505 35253464 10.1161/JAHA.121.024505 PMC9075270

[11] Juillière Y, Barbier G, Feldmann L, Grentzinger A, Danchin N, Cherrier F (1997) Additional predictive value of both left and right ventricular ejection fractions on long-term survival in idiopathic dilated cardiomyopathy. Eur Heart J 18:276–280. 10.1093/oxfordjournals.eurheartj.a015231 9043845 10.1093/oxfordjournals.eurheartj.a015231

[12] McDonagh TA, Metra M, Adamo M, Gardner RS, Baumbach A, Böhm M, Burri H, Butler J, Čelutkienė J, Chioncel O et al (2021) 2021 ESC Guidelines for the diagnosis and treatment of acute and chronic heart failure. Eur Heart J 42:3599–3726. 10.1093/eurheartj/ehab368 34447992 10.1093/eurheartj/ehab368

[13] Greene SJ, Bauersachs J, Brugts J, Ezekowitz JA, Lam CSP, Lund LH, Ponikowski P, Voors AA, Zannad F, Zieroth S et al (2023) Worsening heart failure: nomenclature, epidemiology, and future directions. J Am Coll Cardiol 81:413–424. 10.1016/j.jacc.2022.11.023 36697141 10.1016/j.jacc.2022.11.023

[14] Schulz-Menger J, Bluemke DA, Bremerich J, Flamm SD, Fogel MA, Friedrich MG et al (2020) Standardized image interpretation and post-processing in cardiovascular magnetic resonance-2020 update: society for cardiovascular magnetic resonance (scmr): board of trustees task force on standardized post-processing. J Cardiovasc Magn Reson 22(1):19. 10.1186/s12968-020-00610-6 32160925 10.1186/s12968-020-00610-6 PMC7066763

[15] Barreiro-Pérez M, Curione D, Symons R, Claus P, Voigt JU, Bogaert J (2018) Left ventricular global myocardial strain assessment comparing the reproducibility of four commercially available CMR-feature tracking algorithms. Eur Radiol 28:5137–5147. 10.1007/s00330-018-5538-4 29872912 10.1007/s00330-018-5538-4

[16] Liang K, Baritussio A, Palazzuoli A et al (2021) Cardiovascular magnetic resonance of myocardial fibrosis, edema, and infiltrates in heart failure. Heart Fail Clin 17:77–84. 10.1016/j.hfc.2020.08.013 33220888 10.1016/j.hfc.2020.08.013

[17] Zhuang B, Sirajuddin A, Wang S, Arai A, Zhao S, Lu M (2018) Prognostic value of T1 mapping and extracellular volume fraction in cardiovascular disease a systematic review and meta-analysis. Heart fail rev 23(5):723–731. 10.1007/s10741-018-9718-8 29968223 10.1007/s10741-018-9718-8

[18] Hor KN, Gottliebson WM, Carson C, Wash E, Cnota J, Fleck R et al (2010) Comparison of magnetic resonance feature tracking for strain calculation with harmonic phase imaging analysis. JACC Cardiovasc Imagin 3:144–151. 10.1016/j.jcmg.2009.11.006 10.1016/j.jcmg.2009.11.006 20159640

[19] Estes NAM, Saba S (2021) Cardiac magnetic resonance imaging in nonischemic cardiomyopathy. Circulation 143:1374–1376. 10.1161/CIRCULATIONAHA.120.052929 33819073 10.1161/CIRCULATIONAHA.120.052929

[20] Azuma M, Kato S, Kodama S, Hayakawa K, Kagimoto M, Iguchi K, Fukuoka M, Fukui K, Iwasawa T, Utsunomiya D, Kimura K (2020) Relationship between cardiac magnetic resonance derived extracellular volume fraction and myocardial strain in patients with non-ischemic dilated cardiomyopathy. Eur Heart J. 10.1093/ehjci/ehaa946.0239 10.1016/j.mri.2020.09.004 32898651

[21] Pi SH, Kim SM, Choi JO, Kim EK, Chang SA, Choe YH et al (2018) Prognostic value of myocardial strain and late gadolinium enhancement on cardiovascular magnetic resonance imaging in patients with idiopathic dilated cardiomyopathy with moderate to severely reduced ejection fraction. J Cardiovasc Magn Reson 20:36. 10.1186/s12968-018-0466-7 29898740 10.1186/s12968-018-0466-7 PMC6001169

[22] Ji M, Wu W, He L, Gao L, Zhang Y, Lin Y et al (2022) Right ventricular longitudinal strain in patients with heart failure. Diagnostics 12:445. 10.3390/diagnostics12020445 35204536 10.3390/diagnostics12020445 PMC8871506

[23] Park JH, Park JJ, Park JB et al (2018) Prognostic value of biventricular strain in risk stratifying in patients with acute heart failure. J Am Heart Assoc 7:e009331. 10.1161/JAHA.118.009331 30371332 10.1161/JAHA.118.009331 PMC6404866

[24] Raafs AG, Vos JL, Henkens MT, Slurink BO, Verdonschot JA, Bossers D, Roes K, Gerretsen S, Knackstedt C, Hazebroek MR, Nijveldt R (2022) Left atrial strain has superior prognostic value to ventricular function and delayed-enhancement in dilated cardiomyopathy. JACC Cardiovasc Imaging 15(6):1015–1026. 10.1016/j.jcmg.2022.01.016 35680209 10.1016/j.jcmg.2022.01.016

[25] Cojan-Minzat BO, Zlibut A, Muresan ID, Orzan RI, Cionca C, Horvat D, David L, Visan AC, Florea M, Agoston-Coldea L (2021) Left atrial geometry and phasic function determined by cardiac magnetic resonance are independent predictors for outcome in non-Ischaemic dilated cardiomyopathy. Biomedicine 9(11):1653 10.3390/biomedicines9111653 PMC8615501 34829882

[26] Li Y, Yuanwei X, Tang S, Jiang X, Li W, Guo Ji, Yang F, Ziqian X, Sun J, Han Y, Zhu Y, Chen Y (2021) Left atrial function predicts outcome in dilated cardiomyopathy: fast long-axis strain analysis derived from MRI. Radiology 302:72–81. 10.1148/radiol.2021210801 34698565 10.1148/radiol.2021210801

[27] Li Y, Guo J, Li W, Xu Y, Wan K, Xu Z et al (2022) Prognostic value of right atrial strain derived from cardiovascular magnetic resonance in non-ischemic dilated cardiomyopathy-journal of cardiovascular magnetic resonance. J Cardiovasc Magn Reson 24:54. 10.1186/s12968-022-00894-w 36352424 10.1186/s12968-022-00894-w PMC9648034

